# The INDEPTH Data Repository: An International Resource for Longitudinal Population and Health Data From Health and Demographic Surveillance Systems

**DOI:** 10.1177/1556264615594600

**Published:** 2015-07

**Authors:** Kobus Herbst, Sanjay Juvekar, Tathagata Bhattacharjee, Martin Bangha, Nidhi Patharia, Titus Tei, Brendan Gilbert, Osman Sankoh

**Affiliations:** 1 INDEPTH Network, Accra, Ghana; 2 The Africa Centre for Health and Population Studies,UKZN, Durban, South Africa; 3 KEM Hospital Research Centre, Pune, India; 4 University of the Witwatersrand, Johannesburg, South Africa; 5 Hanoi Medical University, Vietnam

**Keywords:** data sharing, health and demographic surveillance, data repository, research data management, metadata

## Abstract

The International Network for the Demographic Evaluation of Populations and Their Health (INDEPTH) is a global network of research centers that conduct longitudinal health and demographic evaluation of populations in low- and middle-income countries (LMICs) currently in 52 health and demographic surveillance system (HDSS) field sites situated in sub-Saharan Africa (14 countries), Asia (India, Bangladesh, Thailand, Vietnam, and Indonesia), and Oceania (Papua New Guinea). Through this network of HDSS field sites, INDEPTH is capable of producing reliable longitudinal data about the lives of people in the research communities as well as how development policies and programs affect those lives. The aim of the INDEPTH Data Repository is to enable INDEPTH member centers and associated researchers to contribute and share fully documented, high-quality datasets with the scientific community and health policy makers.

## Introduction

The International Network for the Demographic Evaluation of Populations and Their Health (INDEPTH) is a global network of research centers that conduct longitudinal health and demographic evaluation of populations in low- and middle-income countries (LMICs). INDEPTH member health and demographic surveillance systems (HDSSs) contribute annually updated individual-level datasets (core micro dataset) representing basic demographic events (births, deaths, migrations) and person years under surveillance (exposure). Data are collected in defined geographic areas through regular household visits following an initial census using either paper-based or electronic questionnaires. In addition, datasets from multi-center studies conducted in INDEPTH member HDSSs are shared on the repository. Every dataset in the repository is documented using an internationally accepted metadata standard by the Data Documentation Initiative (DDI). Digital object identifiers (doi) are assigned to all the datasets to aid citation.

The INDEPTH Data Management Programme (IDMP, formerly known as iSHARE) assists centers and network studies with dataset extraction, harmonization, quality control, and documentation, and administers the INDEPTH Data Repository (http://www.indepth-ishare.org) aimed at sharing the INDEPTH data globally.

INDEPTHStats is a website for visualizing key demographic indicators based on the core micro datasets in the repository (http://www.indepth-ishare.org/indepthstats/).

## Key Characteristics of the INDEPTH Data Repository

- The INDEPTH Data Repository is a unique resource for complete and detailed longitudinal public health data from geographically defined populations in LMICs.
- The datasets available on the repository will grow over time as more member centers provide core micro datasets, and more studies conducted in the INDEPTH network share data on the INDEPTH Data Repository.
- At the time of publication, the core micro datasets on the repository have data from 25 centers representing 2 million individuals and 24 million person years of observation.
- The repository contains the largest dataset on cause specific mortality in LMICs ever published.
- Data contained in the repository have been used to describe the population impact of major infectious diseases such as malaria, HIV, and TB, as well as the impact of relevant interventions.
- The data are also suitable for quantifying millennium development goals (MDGs; for example, MDG 5 and 6) in select populations.

## Data Resource Basics

INDEPTH Network (Sankoh & Byass, 2012) developed the INDEPTH Data Repository to make data originating from the longitudinal surveillance conducted by its member HDSSs available to the scientific community. Each HDSS maintains a dynamic population cohort, which is regularly surveyed to build up a longitudinal database of individuals and social units in the surveillance areas. INDEPTH encourages its members to release a snapshot of this database on an annual basis as a core micro dataset on the repository. These datasets are in a standard format (Sankoh & Byass, 2012) and represent the basic demographic events (births, deaths, migrations) and person years under surveillance (exposure) of the complete HDSS population. In addition, datasets from multi-center studies conducted in INDEPTH member HDSSs are shared on the repository. Examples of such datasets include the cause-specific mortality dataset (Streatfield et al., 2014) and soon to be released datasets from the Migration and Urbanization working group (Gerritsen et al., 2013). Table 1 lists the HDSSs that have released core micro datasets on the repository.

**Table 1.** HDSSs That Have Contributed Core Micro Datasets to the INDEPTH Data Repository.

Network HDSS^a^	Country	Period (from-to)	Current population^b^	Total population^c^	Person years
Ouagadougou (BF041)	Burkina Faso	2009-2012	82,124	121,728	381,195
Taabo (CI011)	Côte d’Ivoire	2009-2012	40,625	62,331	194,054
Gilgel Gibe (ET021)	Ethiopia	2006-2012	58,150	73,825	402,338
Kilite Awlaelo (ET031)	Ethiopia	2010-2012	64,549	74,794	210,738
Kersa (ET041)	Ethiopia	2008-2012	60,262	67,247	294,872
Dabat (ET051)	Ethiopia	2009-2012	43,782	51,908	186,398
Vadu (IN021)	India	2009-2012	138,072	189,075	582,408
Kilifi (KE011)^d^	Kenya	2003-2012	256,317	547,542	3,937,309
Kisumu (KE021)^d^	Kenya	2003-2012	246,403	427,815	2,796,712
Nairobi (KE031)	Kenya	2002-2012	66,428	190,862	1,273,514
Mbita (KE041)	Kenya	2009-2012	59,790	76,212	260,878
Kombewa (KE051)	Kenya	2011-2012	140,312	145,814	194,338
Karonga (MW011)	Malawi	2003-2012	36,739	59,774	430,316
IRD-Mlomp (SN012)	Senegal	1990-2012	8,416	16,705	269,617
IRD–Niakhar (SN013)	Senegal	1983-2012	42,592	77,715	1,429,640
Agincourt (ZA011)	South Africa	1992-2012	98,923	210,384	2,696,271
Dikgale (ZA021)	South Africa	1995-2012	37,182	46,851	281,920
The Africa Centre for Health and Population Studies (ZA031)	South Africa	2000-2012	71,813^e^	138,964	1,377,854
Ifakara Health Institute–Ifakara Rural (TZ011)	Tanzania	1997-2012	130,256	279,450	2,415,177
Ifakara Health Institute–Rufiji (TZ012)	Tanzania	1998-2012	93,441	172,144	1,554,215
Ifakara Health Institute–Ifakara Urban (TZ013)	Tanzania	2008-2012	40,380	66,722	267,860
Magu (TZ021)	Tanzania	1994-2012	33,058	105,632	1,117,087
Iganga/Mayuge (UG011)	Uganda	2005-2012	77,113	123,052	710,676
Hanoi Medical University–Filabavi (VN012)	Vietnam	1999-2012	51,817	75,839	865,450
Chililab (VN021)	Vietnam	2004-2012	53,399	74,491	547,633
Total			2,031,943	3,476,876	24,678,471

*Note.* HDSS = Health and Demographic Surveillance System; INDEPTH = International Network for the Demographic Evaluation of Populations and Their Health. IRD = L’Institut de recherche pour le développement.

a The text in brackets is the center code that identifies the HDSS in the dataset.

b Population under observation at the end of the reporting period.

c The total number of individuals who have contributed to the person years of exposure.

d Awaiting data use approval for placement on the repository.

e Resident population only.

All datasets are documented using a standard DDI (Vardigan, Heus, & Thomas, 2008) document template summarized in Table 2.

**Table 2.** Standard Metadata Template for INDEPTH Data Repository Datasets.

Section	Description
Document description	This section contains information about the metadata itself, which is the DDI document used to describe the dataset.
Title	Contains the full authoritative title of the DDI document. Equivalent to Dublin Core Title.
DDI document ID number	A unique identifier for the DDI documentation file. The document ID is constructed as follows: DDI.DOI where • DOI is Digital Object Identifier (doi) suffix associated with this dataset, please refer to the study ID number for a description of the format of the doi.
Metadata producer	Name of the person(s) or organization(s) who documented the dataset.
Date of production	Date the marked-up document was produced (not distributed or archived). Equivalent to Dublin Core Date.
DDI document version	A version number and description of this version of the document
Version notes	Additional information regarding the version, in particular to indicate what makes a new version different from its predecessor.
Study description	This section contains information about the study or data collection that is the source of the dataset/s being documented and shared. This section includes information about how the study should be cited, who collected or compiled the data, who distributes the data, keywords about the content of the data, summary (abstract) of the content of the data, data collection methods and processing
Identification	Citation for the data collection/study described by the metadata.
Title	Contains the full authoritative title of the data collection. The title will in most cases be identical to the Document Title (see above)
ID number	The ID number of a dataset is a unique number that is used to identify that dataset. This number forms the basis of the doi associated with the dataset and is identical to the suffix of the doi. It is of the form: INDEPTH.CCNNS.N.VV, where • CNNS is the INDEPTH Member site code: CC the ISO 3166-1 alpha-2 code of the country where the site is situated. NN is a sequential number uniquely identifying an INDEPTH member centre within the country. S is a sequential character uniquely identifying the geographical surveillance site within the centre. • N is the dataset abbreviated name, e.g., CMD2011 for the core micro dataset containing data up to the end of 2011. • VV is the version number of the dataset of the form vN, where v is the literal “v” and N is a sequential version number.
Study type	A broad category defining the type survey or study, e.g., demographic surveillance, sample survey, clinical trial, etc.
Series information	If the dataset is part of network program or working group the name of the programme or working group.
Version	Identify substantive changes to the dataset/s.
Description	A version number followed by a version label.
Production date	The date of this version.
Notes	Additional information regarding the version, in particular to indicate what makes the new dataset different from its predecessor.
Overview	
Abstract	A summary describing the purpose, nature, and scope of the data collection, special characteristics of its contents, major subject areas covered, and what questions the PIs attempted to answer when they conducted the study.
Kind of data	The type of data included in the dataset
Unit of analysis	Basic unit(s) of analysis or observation that the study describes
Description of scope	A description of the themes covered by the survey. It can be viewed as a summary of the modules that are included in the questionnaire.
Topic classifications	The classification field indicates the broad substantive topic(s) that the data cover. The INDEPTH Data Repository makes use of Medical Subject Headings (MeSH) as a controlled vocabulary.
Coverage	Information about a study’s chronological and geographic coverage
Country	Indicates the country or countries covered in the dataset.
Geographic coverage	Information on the geographic coverage of the data. Include the total geographic scope of the data, and any additional levels of geographic coding provided in the variables. Maps to Dublin Core Coverage.
Universe	A description of the population covered by the data in the file; the group of persons or other elements that are the object of the study and to which the study results refer. Age, nationality, and residence commonly help to delineate a given universe, but any of a number of factors may be involved, such as age limits, sex, marital status, race, ethnic group, nationality, income, etc.
Producers and sponsors	
Investigators	The persons, corporate body, or agency responsible for the data collection’s substantive and intellectual content.
Other producers	This field is provided to list other interested parties and persons that have played a significant but not the leading technical role in implementing and producing the data.
Funding	The source(s) of funds for production of the data collection.
Other acknowledgments	This mandatory field is used to acknowledge the data managers involved in producing the dataset.
INDEPTH member center	The INDEPTH member center/site of origin. If multi-centre datasets are released as a single unit, then this field will be set to INDEPTH Network.
Sampling	
Sampling procedure	The type of sample and sample design used to select the survey respondents to represent the population.
Response rates	The percentage of sample members who provided information.
Data collection	
Dates of collection	Contains the date(s) when the data were collected. Provide details of the start and end date of each data collection.
Time periods	The time periods covered by the data, not the dates of coding or making documents machine-readable or the dates the data were collected.
Frequency of data collection	If the data were collected at more than one point in time, the frequency with which the data were collected. In the case of demographic surveillance sites the number of data collection rounds per year.
Mode of data collection	The method used to collect the data
Notes on data collection	Used to describe noteworthy aspects of the data collection situation. Include information on factors such as cooperativeness of respondents, duration of interviews, number of call-backs, etc.
Questionnaires	The questionnaire(s) used for the data collection.
Data collectors	Information regarding the persons and/or agencies that took charge of the data collection
Supervision	Information on the oversight of the data collection
Data processing	
Data editing	Information on how the data were treated or controlled for in terms of consistency and coherence
Other processing	Information as possible on the data entry design, including details such as: • Preparation of the list of dwellings and census forms for the surveillance round. • How document control was conducted to ensure all census forms were completed. • How data entry took place. What software was used and how many data entry operators where there. • What data quality checking was done on the forms, prior to data entry, by the data entry program during data entry, and in the database itself?
Data appraisal	
INDEPTH data quality metrics	A listing of the INDEPTH quality metrics (provided in the controlled vocabulary) and the measured value of the quality metric.
Data access	
Access authority	The contact person or entity to gain authority to access the data. This field is only applicable if the data have restricted access. Most datasets have direct access and can be downloaded without requesting special permission.
Access conditions	Access to INDEPTH Network data is governed by the INDEPTH Data Access and Sharing policy
Citation requirement	The way that the dataset should be referenced when cited in any publication. Includes a DOI to must be quoted when the dataset is cited.
Disclaimer and copyright	Information regarding responsibility for uses of the data collection and the copyright statement for the data collection.
File description	Consists of information about the particular data file containing numeric and/or numeric + textual information. The data fingerprint of the data file is included as part of this metadata.
Variable description	Consists of elements allowing for detailed descriptive information about each variable in the dataset. This includes information about response and analysis units, question text, interviewer instructions, universe, valid and invalid data ranges, derived variables, and summary statistics

*Note.* INDEPTH = International Network for the Demographic Evaluation of Populations and Their Health; DDI = Data Documentation Initiative.

### Dataset Production Support

Datasets hosted on the INDEPTH Data Repository follow a standard procedure to extract, harmonize, quality assure, and document the data. This process is facilitated by an IDMP support team and the provision of a standard all-in-one computer hardware and software environment called the “Centre-in-a-Box” (CiB).

1. **IDMP Support Team**. Two support nodes have been established, one at the Africa Centre for Health and Population Studies in Umkhanyakude, South Africa, and a second at the KEM Research Centre in Vadu, India. The nodes are supported by the INDEPTH Secretariat who acts as the liaison with INDEPTH member centers. The nodes host a team of three data managers, a data librarian, and a computer systems engineer. The team conducts workshops, responds to support calls lodged with IDMP help desk, and assists participating centers with the extraction and documentation of datasets hosted on the repository.
2. **CiB**. INDEPTH member HDSS data managers are trained in the use of the CiB, and each HDSS receives a CiB to use for dataset production and documentation. The CiB is portable, and data managers or analysts can carry the CiB to data analysis workshops. The CiB consists of the following components:
  - Portable mini server hardware. The hardware hosts an operating environment or hypervisor (http://en.wikipedia.org/wiki/Hypervisor) that supports the virtual operating environments needed for dataset production.
  - Database server. One of the virtual operating environments on the CiB hardware hosts a database system that replicates the operational database of the HDSS to facilitate easy transfer of data from the operational system to the analytical dataset production environment. HDSS uses a variety of database systems, and this arrangement assists in developing common data extraction procedures although database systems may differ from site to site.
  - Data manager workstation. The second virtual operating environment is used to host the software required to prepare and document the datasets that will eventually be shared on the repository. The following free software programs are used:
    - Pentaho Data Integration (Community Edition; Pentaho Corporation, 2014). This program is used to extract data from the different underlying database systems, transform the data into a standard format, and load the data into the repository (Extraction, transformation and loading [ETL]). As far as possible, common ETL scripts are used to ensure consistent processing of the data and to reduce the burden of developing center-specific programming.
    - Nesstar Publisher (Norwegian Social Science Data Services, 2013). This program is an editing tool used to prepare the DDI compliant metadata that documents each dataset on the repository.
  - System server. The third virtual operating environment hosts server components that manage the CiB environment, including system security, the shared file system, and a web server. The key software programs are as follows:
    - Zentyal (Zentyal, 2013). A Linux-based server that manages network security, user authentication, and a shared file system for the CiB.
    - Microdata Cataloguing Tool (National Data Archive, [NADA]); The World Bank, 2013). The CiB provides a local implementation of the World Bank-developed Microdata Cataloguing Tool. This local instance is used to view the data documentation prior to uploading the documentation to the network repository. The INDEPTH Repository is also based on this web-based content management application.

### Dataset Production Process

**Figure 1.**
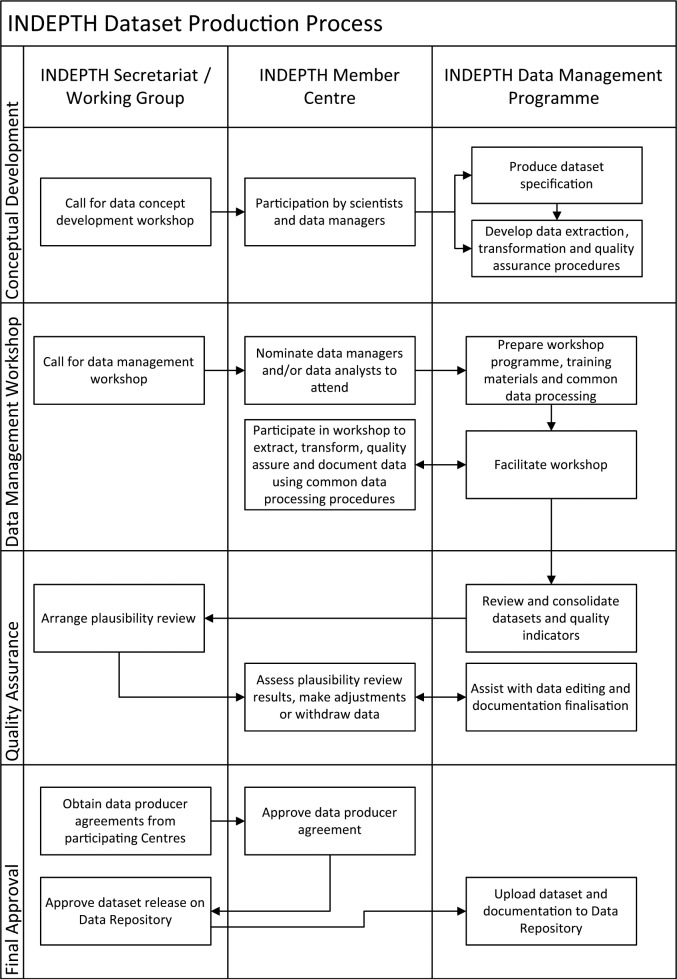
Dataset production process.

With the exception of the core standard micro dataset, which represents the basic demographic events obtained from the HDSS operations, datasets originate from multi-center research or data analysis efforts by scientists from INDEPTH member HDSSs around a common research theme or question. The dataset production process generally follows a standard process to ensure the consistency and quality of the datasets hosted on the INDEPTH Data Repository (Figure 1).

1. **Conceptual Development**. When the need to develop standardized analytical datasets arises from the research or data analysis efforts of INDEPTH member HDSSs, the first step is to develop a common data specification. The specification contains the standard layout of the data file(s) and definitions for all the variables. Eligible populations, time periods, and data measures are also standardized. The IDMP staff then develop standard data extraction, transformation, and quality assurance procedures for the dataset with input from the participating researchers.
2. **Data Management Workshop**. The actual dataset production takes place during joint data management workshops attended by data managers and analysts from the participating INDEPTH HDSSs. The INDEPTH Secretariat issues a call for participants to eligible INDEPTH member HDSSs. The workshop is facilitated by IDMP staff and where necessary workshop attendees receive training in using the dataset production tools (CiB) and applying the common data processing procedures. The dataset production skills acquired by the data managers at the workshop are of general benefit to them when they return to their respective centers. Data quality metrics are calculated for all the datasets and reviewed during the workshop by all participants. Minimum acceptable levels for the data quality metrics are agreed to, and datasets are not accepted for further processing if they fail to reach these levels. Data anonymization (masking data by retaining internal mapping to the original identifier) and identity disclosure risk assessment are also applied to the datasets at this stage.
3. **Quality Assurance**. If datasets (or indicators derived from the datasets) have passed the minimum data quality metrics, summary indicators derived from the datasets are provided to the INDEPTH Secretariat for expert plausibility review. Plausibility review reports are fed back to the participating HDSSs, and a final decision is made jointly with the HDSS regarding the suitability of their dataset for inclusion on the INDEPTH Data Repository. The IDMP staff are not involved in this decision.
4. **Final Approval**. The INDEPTH Secretariat obtains signed data producer agreements from the participating HDSSs with datasets suitable for inclusion on the data repository. The data producer agreements are prescribed by the INDEPTH Data Access and Sharing Policy (Sankoh & Byass, 2012) and confirm that the HDSS (and associated investigators) agree to the hosting of their dataset on the repository at a specified data access level (described under resource use). The data producer also confirms that there are no ethical or legal obligations that prevent the use and sharing of the datasets.

## Data Resource Use

The INDEPTH Data Access and Sharing Policy (Sankoh & Byass, 2012) identifies the following levels of access to shared network data:

1. **Open Access.** Except for attribution of origin, no conditions and prior registration are applicable to the use of the data. This level of access is applicable only to aggregated data on the INDEPTHStats website.
2. **Licensed Access**. Registration by the prospective user on the INDEPTH Repository is required, but other than a statement of purpose for which the data will be used and a click-through agreement with the terms of data use, no further approvals are required to access the dataset.
3. **Restricted Licensed Access**. In addition to the requirements for licensed access, there is an additional approval step by the dataset custodian. The prospective data user submits the required information by completing a form on the repository, which is emailed by the repository to the IDMP help desk, who in turn contacts the dataset custodian to provide approval to the request. Once approval is received, the IDMP support team enables access to the dataset for the data user.
4. **Closed Access**. This applies to highly sensitive or individually identifiable data. Such data are normally available to prospective users only through controlled-on-site access and/or in collaboration with the member centers involved. Only the metadata are published on the repository.

The INDEPTH Data Repository uses different terminology to identify these access levels, and the equivalent access levels are tabulated in Table 3.

**Table 3.** INDEPTH Data Access Policy Levels Compared With the Built-In Access Levels on the Repository.

INDEPTH data access policy level	INDEPTH data repository access level	Description
	Data not available	Not applicable
Open access	Direct access data files	The user is not required to be logged into the site and no personal information is collected on the person downloading the data.
Licensed access	Public use data files	The user must be logged in and registered on the site before they are able to download the data. The user is required to agree to a terms of use of the data and the repository keeps a record of who downloads the data.
Restricted licensed access	Licensed data files	Users are required to fill in and submit a detailed application form listing their reasons for wanting access to the data. Once the user submits the application form the system informs the system administrator that an application has been made. For the person to get access to the data, the system administrator needs to review the application and approve it.
Closed access	Data available in an enclave	No data are shared on the repository. Users submit an application to access the data on-site at the submitting INDEPTH member center.
	Data available from external repository	The repository allows for studies and their metadata to be listed on the repository but for a link to be created to another site where the data reside.

*Note*. INDEPTH = International Network for the Demographic Evaluation of Populations and Their Health.

When downloading data from the repository, the user agrees, by accepting the click-through data use agreement, to the following conditions:

1. To not redistribute or sell the data;
2. In the case of multi-site datasets, to not analyze or report on a single site’s data without permission from the site concerned;
3. To not attempt to identify individuals;
4. To not produce links to other datasets that could identify individuals;
5. To cite the source of the data according to the citation requirement provided with the dataset;
6. To provide copies of publications based on the data to INDEPTH;
7. A disclaimer that the original collector of the data, INDEPTH or the relevant funding agencies bear no responsibility for the data’s use or inferences based on it.

The repository records page views by prospective data users as well as dataset downloads. In the case of licensed and restricted licensed access datasets, user details are recorded as well. Table 4 summarizes the region of origin for the 724 downloads that took place between the launch of the repository on July 1, 2013, and at the end of June 2015.

**Table 4.** Dataset Download by Region Between July 1, 2013, and June 29, 2015.

Region	*n*
Africa	225
Asia	110
Europe	171
North America	217
Other	1
Total	724

The INDEPTH Network is registered with DataCite (2014) through the GESIS–Leibniz Institute for the Social Sciences and has been allocated the 10.7796 doi (Paskin, 2008) prefix. All datasets are registered with a unique doi that must be included when the dataset is cited.

A digital fingerprint (Altman & King, 2007) is calculated using an MD5 (Rivest, 1992) hash function for each dataset. This universal numeric fingerprint (UNF) is stored as part of the metadata describing the dataset, and a data user can use the UNF to verify that the data were not intentionally or unintentionally altered.

INDEPTHStats is a website associated with the INDEPTH Data Repository for visualizing key demographic indicators based on the core micro datasets in the repository. This assists prospective data users and policy makers to obtain a quick overview of the information contained in the detailed datasets on the repository without needing to analyze the datasets first.

## Strengths and Weaknesses

### Strengths

- Detailed data from populations without vital registration
- Longitudinal with accurate denominators
- Datasets in easy-to-analyze event history format
- Dataset published in widely supported UTF8 encoded comma separated text files

### Weaknesses

- Available data limited both in scope and number of sites, but will expand in future
- Not representative in the traditional sense, but still very useful in providing insights into population dynamics and health intervention impact
